# RNA Molecular Signature Profiling in PBMCs of Sporadic ALS Patients: HSP70 Overexpression Is Associated with Nuclear SOD1

**DOI:** 10.3390/cells11020293

**Published:** 2022-01-15

**Authors:** Maria Garofalo, Cecilia Pandini, Matteo Bordoni, Emanuela Jacchetti, Luca Diamanti, Stephana Carelli, Manuela Teresa Raimondi, Daisy Sproviero, Valeria Crippa, Serena Carra, Angelo Poletti, Orietta Pansarasa, Stella Gagliardi, Cristina Cereda

**Affiliations:** 1 Genomic and Post-Genomic Unit, IRCCS Mondino Foundation, Via Mondino, 27100 Pavia, Italy; maria.garofalo@mondino.it (M.G.); cecilia.pandini@unimi.it (C.P.); matteo.bordoni@mondino.it (M.B.); daisy.sproviero@mondino.it (D.S.); orietta.pansarasa@mondino.it (O.P.); cristina.cereda@mondino.it (C.C.); 2 Department of Biology and Biotechnology “L. Spallanzani”, University of Pavia, Via Ferrata, 27100 Pavia, Italy; 3 Department of Chemistry, Materials and Chemical Engineering “Giulio Natta”, Politecnico di Milano, Piazza Leonardo da Vinci, 20133 Milan, Italy; emanuela.jacchetti@polimi.it (E.J.); manuela.raimondi@polimi.it (M.T.R.); 4 Neuro-Oncology Unit, IRCCS Mondino Foundation, Via Mondino, 27100 Pavia, Italy; luca.diamanti@mondino.it; 5 Department of Biomedical and Clinical Sciences “L. Sacco”, University of Milan, Via Giovanni Battista Grassi, 20157 Milan, Italy; stephana.carelli@unimi.it; 6 Pediatric Clinical Research Center Fondazione “Romeo ed Enrica Invernizzi”, University of Milano, Via Festa del Perdono, 20122 Milano, Italy; 7 Dipartimento di Scienze Farmacologiche e Biomolecolari (DiSFeB), Università degli Studi di Milano, Via Balzaretti, 20133 Milano, Italy; valeria.crippa@unimi.it (V.C.); angelo.poletti@unimi.it (A.P.); 8 Department of Biomedical, Metabolic and Neural Sciences, University of Modena and Reggio Emilia, Via Giuseppe Campi, 41125 Modena, Italy; serena.carra@unimore.it

**Keywords:** ALS, DNA damage, HSP70, SOD1, transcriptomics

## Abstract

Superoxide dismutase 1 (SOD1) is one of the causative genes associated with amyotrophic lateral sclerosis (ALS), a neurodegenerative disorder. SOD1 aggregation contributes to ALS pathogenesis. A fraction of the protein is localized in the nucleus (nSOD1), where it seems to be involved in the regulation of genes participating in the oxidative stress response and DNA repair. Peripheral blood mononuclear cells (PBMCs) were collected from sporadic ALS (sALS) patients (*n* = 18) and healthy controls (*n* = 12) to perform RNA-sequencing experiments and differential expression analysis. Patients were stratified into groups with “high” and “low” levels of nSOD1. We obtained different gene expression patterns for high- and low-nSOD1 patients. Differentially expressed genes in high nSOD1 form a cluster similar to controls compared to the low-nSOD1 group. The pathways activated in high-nSOD1 patients are related to the upregulation of HSP70 molecular chaperones. We demonstrated that, in this condition, the DNA damage is reduced, even under oxidative stress conditions. Our findings highlight the importance of the nuclear localization of SOD1 as a protective mechanism in sALS patients.

## 1. Introduction

Amyotrophic lateral sclerosis (ALS) is a rare and fatal neurodegenerative disease characterized by the degeneration and loss of cortical, bulbar and spinal motor neurons, culminating in muscle denervation and paralysis. ALS can occur sporadically, without any family history (sALS; 90–95% of patients), while a small percentage of ALS cases are considered familial (fALS; 5–10%). Cu/Zn superoxide dismutase 1 (SOD1), a homodimeric metalloprotein with antioxidant function, has been identified as one of the pathogenic proteins involved in ALS development. It is mainly distributed in the cytoplasm; however, it has also been found in the nucleus, lysosomes and mitochondria [1]. Its mutations correlate with DNA damage and mitochondrial dysfunctions [2,3,4]. All protein products of this mutant gene are capable of misfolding and aggregating intracellularly, causing proteotoxic stresses and possibly toxicity. Indeed, increased cytoplasmatic aggregation, dimer destabilization and oligomerization are all mechanisms proposed for mutant SOD1 toxicity in ALS, and they might not be mutually exclusive [5,6].

The involvement of wild-type SOD1 in sALS cases has been investigated given its reduced expression in lysates from peripheral blood mononuclear cells (PBMCs) [7], which is in discordance with the abnormal upregulation of its transcript [8]. PBMCs have been adopted in several studies on ALS because of the value of proteins expressed in these cells as diagnostic and prognostic biomarker. Within the cells, SOD1 is localized in both the cytoplasm and nucleus, with the two pools of SOD1 having distinct functions. It has been suggested that nuclear SOD1 could also act as a scavenger enzyme in the nucleus to regulate the oxidative stress response [9], while its cytoplasmic retention due to, e.g., genetic mutations or post-translational modifications could enhance cell vulnerability to oxidation and DNA damage [2,10,11]. SOD1 distribution in sALS PBMCs led to the identification of two subgroups of patients: those with high nSOD1 and those with low nSOD1 [12]. Protein relocalization causes a reduction in DNA damage when SOD1 is accumulated as soluble protein within the nucleus (nSOD1), while it is associated with extensive DNA damage when it aggregates in the cytoplasm. In addition, a positive correlation between longer survival and a higher amount of soluble SOD1 in the nucleus was described, and this suggests that SOD1 may exert a protective role against neurodegeneration when located within the nucleus, while aggregated cytoplasmic SOD1 could be impaired in its protective activity and potentially harmful [3,13]. 

Based on these findings, we stratified sALS patients into subgroups according to high or low SOD1 levels in the nucleus of PBMCs. Our aim was to perform whole transcriptome profiling of these two groups of sALS patients and matched controls for the identification of deregulated genes. Starting from RNA deregulation, we extrapolated altered pathways and validated them in vitro to confirm RNA-sequencing analysis results. The results may provide interesting insights on pathways that are directly or indirectly activated by nSOD1 and crucial molecular mechanisms involved in sALS pathogenesis.

## 2. Materials and Methods

### 2.1. Patient Enrollment 

Recruitment resulted in 18 sALS patients and 12 age- and sex-matched healthy controls. All subjects provided written informed consent (Protocol n° 20180034329). Only subjects not affected by any neurological condition or other relevant comorbidities were selected as “healthy controls”. IRCCS Mondino Foundation (Pavia, Italy) conducted clinical and neurological checkups of ALS patients, who were diagnosed with ALS as defined by El Escorial criteria. In addition, genetic screening was conducted to exclude patients carrying mutations in FUS, TARDBP, SOD1, VCP, ANG and C9orf72 genes. The control subjects were recruited at the Transfusional Service and Centre of Transplantation Immunology, IRCCS Foundation San Matteo, (Pavia, Italy). The Ethical Committee of the IRCCS Mondino Foundation (Pavia, Italy) approved the study protocol to obtain peripheral blood from patients and controls. All experiments were performed in accordance with relevant guidelines and regulations.

### 2.2. PBMC Isolation from Blood Samples

Centrifugation was used for PBMC preparation. Peripheral blood was layered (density = 1.077) with Ficoll-Histopaque (Sigma-Aldrich, St. Louis, MO, USA ) and centrifuged at 1800 rpm for 30 min. Cells used for RNA extraction were previously selected after assessing viability through trypan blue exclusion test.

### 2.3. Subcellular Fractionation

The method of Schreiber and colleagues [14] was used for the subcellular fractionation of PBMCs, with minor modifications. Ice-cold hypotonic lysis buffer (10 mM HEPES, pH 7.9, 10 mM KCl, 0.1 mM EDTA, 1 mM dithiothreitol, 0.5 mM phenylmethylsulfonyl fluoride, 1% protease and phosphatase inhibitor cocktail) was used to resuspend cells. Then, they were incubated on ice for 25 min for swelling, after which 25 µL of 10% Nonidet NP-40 (Sigma-Aldrich) was added. Samples were vortexed and centrifuged at full speed to separate the supernatant (containing cytoplasmic proteins) and pellets (containing nuclear proteins). Ice-cold hypertonic nuclear extraction buffer (20 mM HEPES, pH 7.9, 0.4 M NaCl, 1 mM EDTA, 1 mM dithiothreitol, 1 mM phenylmethylsulfonyl fluoride, 1% protease and phosphatase inhibitor cocktail) was used to resuspend nuclear pellets, which were incubated on ice for 20 min with agitation. The nuclear extracts were then centrifuged at the maximum speed for 5 min at 4 °C to collect supernatant containing the nuclear proteins. Both cytoplasmic and nuclear extracts were stored at −80 °C until use. 

### 2.4. Western Blotting Analysis

Western blotting analysis was performed by SDS–polyacrylamide gel electrophoresis (SDS–PAGE). A 12.5% SDS–PAGE gel (Bio-Rad, Hercules, CA, USA) was used for loading 30 µg of proteins, both cytoplasmic and nuclear. Using a liquid transfer apparatus (Bio-Rad), samples were transferred to a nitrocellulose membrane (Bio-Rad) after electrophoresis. For blocking nonspecific protein binding sites, nitrocellulose membranes were treated with a blocking solution (5% nonfat dry milk in TBS-T buffer, 10 mM Tris-HCl, 100 mM NaCl, 0.1% Tween, pH 7.5). Incubation with primary antibodies was conducted overnight at 4 °C (SOD1 (sc-11407), PCNA (sc-56), HSF1 (sc-17757), all from Santa-Cruz (Dallas, TX, USA); HSP70 (ab2787), HSPH1 (ab109624), pHSF1 (ab76076), all from Abcam (Cambridge, UK); GAPDH (GTX100118), GeneTex, Irvine, CA, USA). Immunoreactivity was detected using donkey anti-rabbit or anti-mouse secondary peroxidase-conjugated antibody (GE Healthcare, Chicago, IL, USA), and bands were visualized using enhanced chemiluminescence detection kit (ECL Select, GE Healthcare). Stripping solution (mercaptoethanol, 2% SDS, and 62.5 mM Tris/HCl, pH 6.7) was used for removing both primary and secondary antibodies from the membrane and then processed as described above. ImageJ software version number 1.51, available online: http://rsb.info.nih.gov/ij/ (accessed on 2 May 2018) was used for performing densitometric analysis of the bands, and statistical analysis was performed using one-way ANOVA (Kruskal–Wallis) and the Bonferroni post-test for all possible test pairings using Prism GraphPad 8.0.2 software (GraphPad Software, San Diego, CA, USA). Two-tailed *p-*values with 95% confidence intervals were computed, and *p <* 0.05 was considered statistically significant.

### 2.5. Selection of a Cut-Off Value

The distribution of the values obtained from Western blotting corresponding to nuclear SOD1 was analyzed, and a bell-shaped distribution was observed in CTRL, whereas a bimodal distribution was observed in sALS. Because the median value of the curve with a normal distribution (mean = 1.46) was located at the highest frequency point between the two distribution peaks, we decided to use this value as an arbitrary cut-off. Similar approaches have been reported in the literature [12,15].

### 2.6. RNA Extraction 

Trizol^®^ reagent (Life Science Technologies, Waltham, MA, USA) was used for total RNA isolation from PBMCs. The manufacturer’s specifications were followed. A Nanodrop ND-100 spectrophotometer (Nanodrop Technologies, Wilmington, DE, USA) was used for RNA quantification, and quality was checked with a 2100 Bioanalyzer (Agilent, Santa Clara, CA, USA). 

### 2.7. Library Preparation for RNA-seq and Bioinformatic Data Analysis

Starting from 500 ng of total RNA and using Illumina TruSeq Stranded RNA Library Prep, version 2, Protocol D (Illumina, San Diego, IL, USA), cDNA libraries were prepared. Total RNA was fractionated for rRNA depletion, as this is not part of the research focus and causes a reduction in transcript coverage. Libraries were prepared as in Gagliardi et al. [16]. 

The quality of each library was assessed by 4200 Tape Station with a “DNA High sensitivity” assay (Agilent). Libraries were fluorometrically quantified using High Sensitivity dsDNA assay with a Qubit device (Life Technologies). The sequencing step was performed with NGS technologies using Illumina Genome Analyzer and the NextSeq 500/550 High Output v2.5 kit (150 cycles) (Illumina), processed on Illumina NextSeq 500. FastQ files were generated via Illumina bcl2fastq2, version 2.17.1.14. available online: http://support.illumina.com/downloads/bcl-2fastq-conversion-software-v217.html (accessed on 2 May 2018 starting from raw sequencing reads produced by Illumina NextSeq sequencer. The number of detected transcripts for coding and noncoding RNAs was evaluated separately for each sample. Gene and transcript intensities were computed using STAR/RSEM software [17] using GRCh38 (Gencode release 27) as a reference and the “stranded” option. Differential expression analysis for mRNA was performed using the R package EBSeq [18], while for lncRNA, the R package DESeq was used [19]. Transcripts with |log2(disease sample/healthy control)| ≥ 1 and FDR ≤ 0.1 were considered differentially expressed and retained for further analysis. 

### 2.8. Pathway Analysis

Gene enrichment analysis was performed on coding genes [20]. A Gene Ontology (GO) analysis was conducted for biological processes, cellular components and molecular function, while KEGG pathway analysis (Kyoto Encyclopedia of Genes and Genomes available online: https://www.genome.jp/kegg/pathway.html; accessed on 2 May 2018) and WikiPathways (available online: https://www.wikipathways.org/index.php/WikiPathways; accessed on 2 May 2018) were conducted for pathway analysis. For this investigation, we used the enrichR web tool (accessed on 10 December 2019) [21,22].

### 2.9. Real-Time PCR

Using the online Primer 3.0 tool for primer design, we selected PCR oligonucleotides for genes pairs (Supplementary Table S4). Starting from 500 ng of total RNA, cDNAs were prepared using iScript™ Reverse Transcription Supermix for RT-qPCR (Bio-Rad). qPCR reactions included 200 nM of each oligonucleotide, 7.5 μL of iQ SYBR Green Supermix (Bio-Rad), and 1 μL of cDNA template (or water control). Cycling conditions using a Bio-Rad iQ5 real-time thermocycler were 5 min denaturation at 95 °C, followed by 40 cycles of 95 °C (10 s) and 60 °C annealing (30 s). Cycle threshold (Ct) values were normalized with GAPDH, and fold expression differences were determined using the 2ΔCt method. The significance of gene expression changes relative to controls was analyzed using one-way ANOVA (Kruskal–Wallis) and the Bonferroni post-test for all possible test pairings using Prism GraphPad 8.0.2 software (GraphPad Software). Two-tailed *p-*values with 95% confidence intervals were computed, and *p <* 0.05 was considered statistically significant. 

### 2.10. Comet Assay

For the DNA damage study, the comet assay was performed in PBMCs of controls and high-nSOD1 and low-nSOD1 sALS patients. PBMCs were treated with 500 μM H_2_O_2_ or with 500 μM H_2_O_2_ (Sigma-Aldrich) + 50 μM VER (Sigma-Aldrich) (HSP70 inhibitor) followed by a 30 min stress recovery phase.

Approximately 1 × 10^4^ cells were used for the comet assay. Cells were resuspended in 0.75% low-melting-point agarose (Sigma-Aldrich) and placed onto microscope slides coated with a layer of 1% agarose in 1X PBS. Nucleus lysis and electrophoresis were conducted as in Bordoni et al. [3]. For visualization and analysis of individual comets using a fluorescence microscope (Axio Imager 2, Zeiss, Jena, Germany), Hoechst dye (Sigma-Aldrich) was added to stain the nucleus. Comet length was measured using CaspLab (1.2.3beta2 version) [23], and values were analyzed using one-way ANOVA (Kruskal–Wallis) and the Bonferroni post-test for all possible test pairings using Prism GraphPad 8.0.2 software (GraphPad Software). Two-tailed *p-*values with 95% confidence intervals were computed, and *p <* 0.05 was considered statistically significant.

### 2.11. Immunofluorescence

A total of 1 × 10^5^ cells were placed on a poly-L-lysine slide (Thermo Fisher Scientific, Waltham, MA, USA) and incubated at 37 °C to allow cell attachment to the slide. Cells were rinsed with 1× PBS and then fixed using a solution of 4% PFA/1× PBS. Fixed cells were washed with 1× PBS and treated with a blocking solution (5% normal goat serum in 0.1% Tween-PBS) for 1 h to block nonspecific protein binding sites. Cells were then incubated ON at 4 °C with primary antibodies: mouse monoclonal anti-histone H3 (trimethyl K27) antibody (ab6002; Abcam). Cells were washed with 1× PBS and incubated at RT for 1 h with secondary antibodies: CFTM 594 goat anti-mouse (Sigma-Aldrich). Both primary and secondary antibodies were prepared in blocking buffer. Finally, samples were washed with 1× PBS, mounted with Prolong^®^ Gold antifade reagent with DAPI (Invitrogen), dried and nail-polished, and images were acquired by confocal microscopy (Olympus Fluoview FV10i, Tokyo, Japan). 

## 3. Results

### 3.1. Whole Transcriptome Analysis in PBMCs of High- and Low-nSOD6 sALS Patients and Healthy Matched Controls 

#### 3.1.1. Classification of sALS Patients with High and Low Nuclear SOD1 

**Figure 1:**
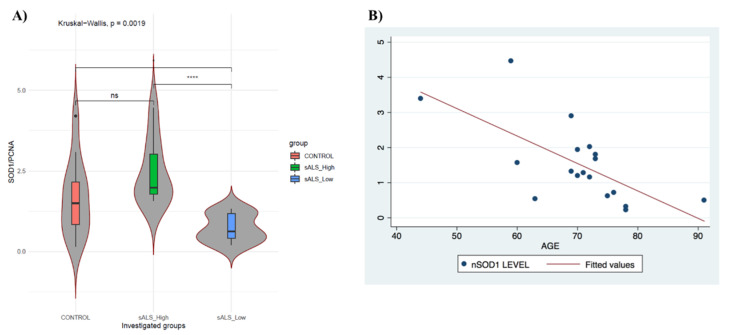
(**A**) PBMCs of sALS patients differ in nSOD1 distribution. Violin plots with boxplot showing distribution of SOD1 in control group (*n* = 12; red), sALS patient group with “high” nSOD1 (*n* = 8; green) and sALS patient group with “low” nSOD1 (*n* = 10; blue). Data were analyzed by Kruskal–Wallis test. **** *p* < 0.005; ns = nonsignificant. Levels of nSOD1 correlate with patients’ age. (**B**) Scatter plot of age vs. nSOD1 levels in patients considered for this work; *p*-value = 0.0021; R^2^ = 0.4456. nSOD1 amount is age-dependent.

To group the sALS patient population and matched controls in relation to nSOD1 levels, we classified high and low nSOD1 by evaluating the levels of SOD1 protein in the nucleus of 12 healthy controls (CTRL) and 18 sALS patients through Western blot analysis (Figure S1). The ratio between SOD1 and nuclear PCNA1 was calculated for normalization (Table 1). The resulting data were used to define a threshold of 1.46 for distinguishing high- and low-nSOD1 groups, as described in Cereda et al. (2013) and Bordoni et al. (2019) [3,12] on a new subset of patients. The population distribution is represented in the violin plot shown in Figure 1A. The mean values of the ratio between SOD1 and PCNA1 in CTRL and high-nSOD1 (*n* = 8) samples were 1.66 and 2.26, respectively. In low nSOD1 (*n* = 10), the mean value of the ratio was 0.78. The density of low nSOD1 values was more homogenous, while those of controls and the high-nSOD1 group showed a similar distribution. 

Since the patients selected for this study were age-heterogeneous, a linear regression model was performed based on the age of patients and the SOD1 concentration in the nucleus (Figure 1B). We found that the levels of nSOD1 strongly decrease with age (*p*-value = 0.0021) in sALS patients. These data may confirm the putative protective role of SOD1 in the nucleus since its expression is lost during aging. SOD1 presence in the nucleus of sALS patients did not have statistically significant correlations with sex, age of onset, disease duration or other clinical parameters.

#### 3.1.2. Whole Transcriptome Analysis in PBMCs of High- and Low-nSOD1 sALS Patients and Healthy Controls 

We performed RNA-seq analysis to investigate the transcriptome profiles in PBMCs of sALS patients (*n* = 18) classified as high nSOD1 (*n* = 8) and low nSOD1 (*n* = 10) based on the nuclear SOD1 ratio and age/sex-matched healthy CTRL (*n* = 12). The number of uniquely mapped reads per sample resulting from the RNA-seq experiment is represented in Figure S2. Genes with |log2(disease sample/healthy donor)| ≥ 1 and false discovery rate ≤ 0.1 were considered differentially expressed and retained for further analysis [24].

We detected differentially expressed (DE) transcripts in PBMCs from sALS patients with high nSOD1 and sALS patients with low nSOD1. Both groups were compared with healthy controls. In low-nSOD1 patients, we found a total of 62 DE genes (Supplementary Table S1), 35 coding genes (20 upregulated and 15 downregulated) and 27 noncoding genes with respect to healthy controls. Among the noncoding genes, 14 were lncRNAs (9 upregulated and 5 downregulated). In high-nSOD1 patients, we found only 25 DE genes versus controls (Supplementary Table S2). There were 15 coding genes (12 upregulated and 3 downregulated) and 10 noncoding genes, of which 4 were lncRNAs (2 upregulated and 2 downregulated). The outcomes of DE analysis are summarized in Table 2, and the biotypes of detected transcripts are represented in Figure S3. 

**Figure 2:**
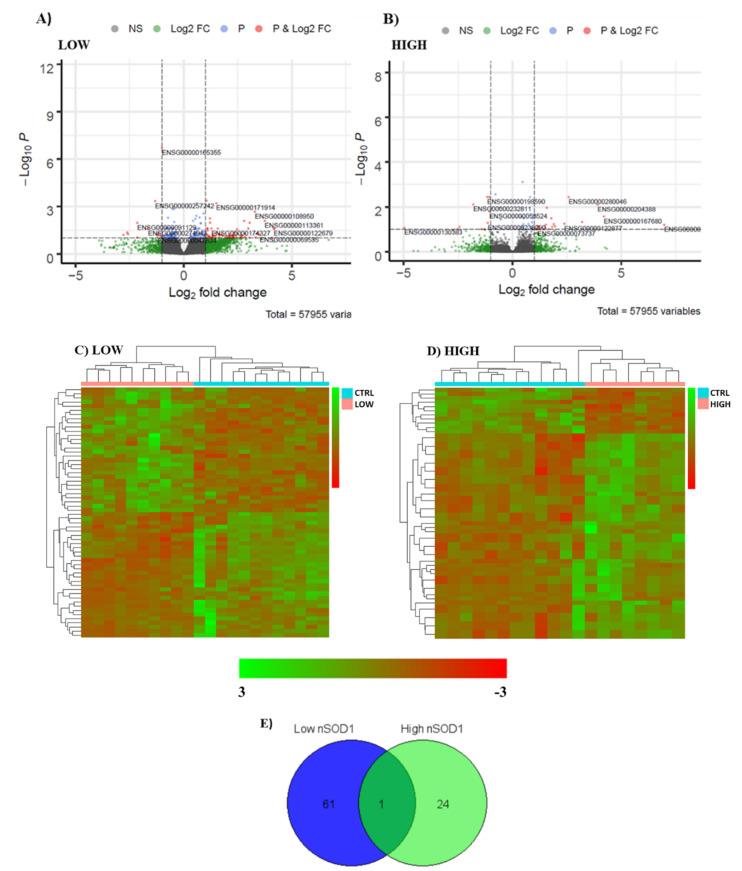
Differential expression analysis in low- and high-nSOD1 PBMCs of sALS patients. Volcano plots. Panel (**A**) shows DE genes in low-nSOD1 patients, while panel (**B**) shows DE genes in high-nSOD1 patients. The expression difference is considered significant for a log2 fold change of ≥1 or ≤−1 (*x*-axis) and for false discovery rate ≤ 0.1 (*y*-axis). Red dots represent significantly up- and downregulated genes that have |log2(fold change)| ≥ 1 and a *p*-value ≤ 0.05. Blue, green and gray dots represent detected DE genes that are not significant, because they do not satisfy both requirements. (NS = nonsignificant; log2FC = satisfying fold change criteria; P: satisfying *p*-value criteria; P and log2FC: satisfying both fold change and *p*-value cut-off). The top 11 DE genes are labeled (Ensembl ID). Heat maps. Expression profiles of differently expressed genes in sALS patients and healthy controls. Panel (**C**) compares RNAs in low-nSOD1 patients (pink bar) and the control samples (light blue bar), while panel (**D**) compares RNAs in high-nSOD1 patients (pink bar) and the control samples (light blue bar). Venn diagram. Differentially expressed genes in common between the two nSOD1 ALS groups (**E**).

We considered the resulting transcripts separately for each group and constructed volcano plots (Figure 2A,B) to highlight all statistically significant DE genes in the two ALS subgroups. 

Heat maps of the top 60 DE genes in both groups are shown in Figure 2C,D. For the low-nSOD1 group, where fewer than 60 DE genes were found, we used all DE genes. It clearly appears that both low- and high-nSOD1 groups have different gene expression profiles compared to healthy controls.

We then compared the DE genes of the two sALS groups to highlight common transcripts. We only detected one common DE gene in the two groups (Figure 2E), which is an antisense lncRNA (MGC16275; fold change (FC) low nSOD1: 1.29; FC high nSOD1: 1.58).

**Figure 3:**
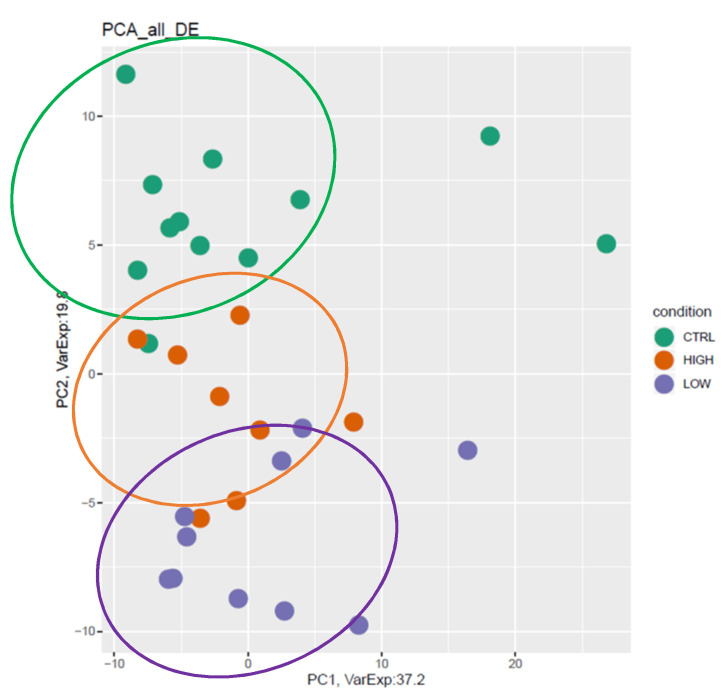
Principal component analysis (PCA) of all differentially expressed genes. PC1: 37.2 (*x*-axis) and PC2: 19.8 (*y*-axis). Both low-nSOD1 (in purple) and high-nSOD1 (in orange) groups are separate from healthy controls (CTRL in green), and interestingly, the high-nSOD1 group is closer to the control group.

We performed a principal component analysis (PCA) of DE genes in the two sALS groups and healthy controls (Figure 3). A neat and gradual division of patients is present. Both low and high groups are separated from healthy controls, and interestingly, the high-nSOD1 group, already described as the “less affected” group with better prognosis [3], is similar to the control group. 

### 3.2. Evaluation of Histone 3 Methylation

**Figure 4:**
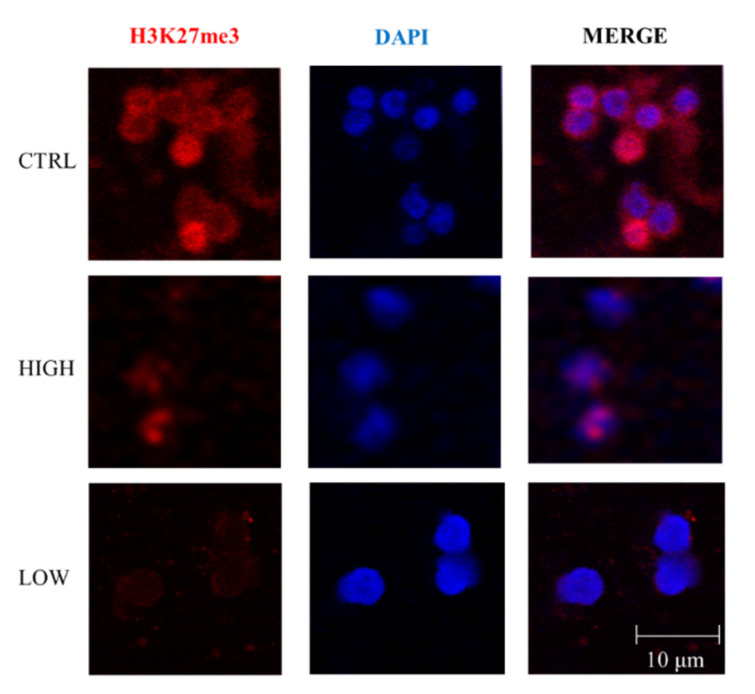
High nSOD1 induces an increase in H3K27 methylation. Representative images of trimethylation of histone 3 on lysine 27 (H3K27me3) investigated through immunofluorescence in PBMCs of controls (*n* = 2) and high-nSOD1 (*n* = 2) and low-nSOD1 (*n* = 2) patients.

Because of the strong difference in gene expression that we observed in the two sALS subgroups and the upregulation of lysine demethylase 4C (KDM4C) in the low-nSOD1 group, we used immunofluorescence to evaluate the trimethylation of histone 3 (H3) in PBMCs. We studied the expression of H3K9me3 and H3K27me3. Only H3K9me3 is a direct substrate of KDM4C [25]. Both of these histone modifications suppress transcription [26]. However, no differences in H3K9me3 were present between the two groups or relative to healthy controls (data not shown). Nevertheless, as shown in Figure 4, in patients with high nSOD1, the amount of H3K27me3 is higher compared with low-nSOD1 groups. This agrees with the lower number of DE genes that we found in the high-nSOD1 group. This evidence highlights a potential link between nSOD1 levels and H3 methylation, with a subsequent effect on the epigenetic regulation of gene expression, leading to altered cellular processes. 

### 3.3. mRNA Pathway Analysis

**Figure 5:**
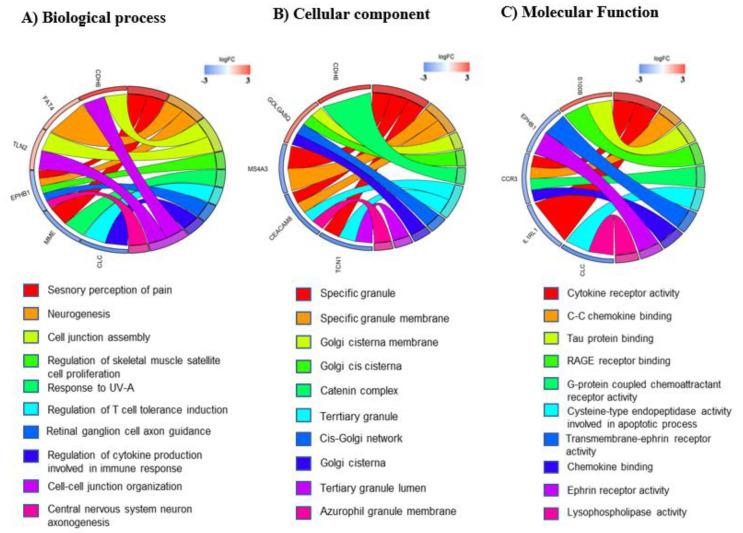

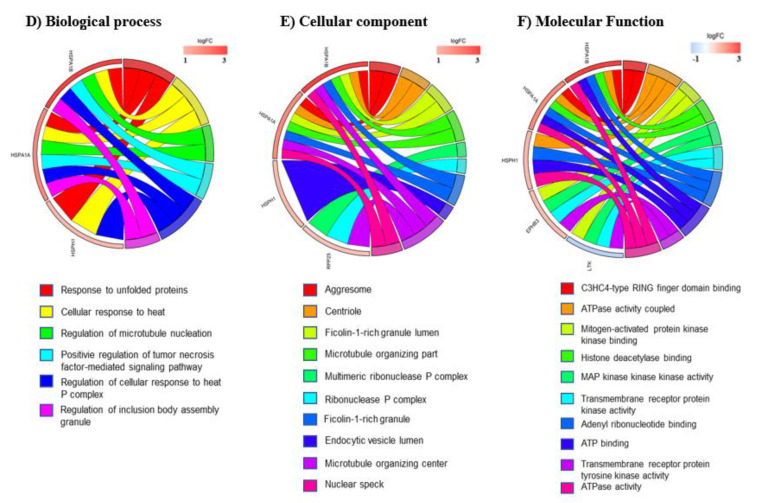
GO chord plot. Chord plot showing significantly enriched GO terms for biological process (**A**,**D**), cellular component (**B**,**E**) and molecular function (**C**,**F**) in low (**A**–**C**) and in high (**D**–**F**) nSOD1. The left of the plot shows the genes contributing to the enrichment, arranged in order of their logFC, which is displayed in descending intensity of red squares for the upregulated genes and blue squares for the downregulated ones. The genes are linked to their assigned terms via colored ribbons.

To further explore the mechanisms underlying the differences observed between the two sALS groups, GO term enrichment analysis for DE genes in high-nSOD1 and low-nSOD1 patients compared to healthy controls was performed for both upregulated and downregulated DE genes together. For low-nSOD1 patients, the enriched GO terms in the biological process category are related to sensory perception of pain, neurogenesis, regulation of skeletal muscle satellite cell proliferation, retinal ganglion cell axon guidance, regulation of cytokine production involved in immune response and central nervous system neuron axonogenesis (Figure 5A). The enriched GO terms in the cellular component category highlight the involvement of granules and the Golgi network (Figure 5B), while those belonging to molecular functions include cytokine receptor activity, Tau protein binding, RAGE receptor binding and Ephrin receptor activity (Figure 5C). 

With regard to DE genes in the high-nSOD1 group, the enriched GO terms in the biological process category include response to unfolded proteins, cellular response to heat and positive regulation of tumor necrosis factor-mediated signaling pathway (Figure 5D). With respect to the cellular component, the most enriched GO terms include aggresome, centriole, microtubule and ribonuclease P complex (Figure 5E). The most enriched GO terms for molecular function are related to RING finger domains, ATPase activity, histone deacetylase binding and transmembrane receptor protein tyrosine kinase activity (Figure 5F). 

We also analyzed the involved pathways resulting from DE genes using both KEGG and WikiPathways databases. The data support differences in pathway activation in the two patient groups and are reported in Supplementary Materials (Figure S4A–D). 

### 3.4. Heat Shock Proteins and DNA Damage Evaluation in PBMCs

**Figure 6:**
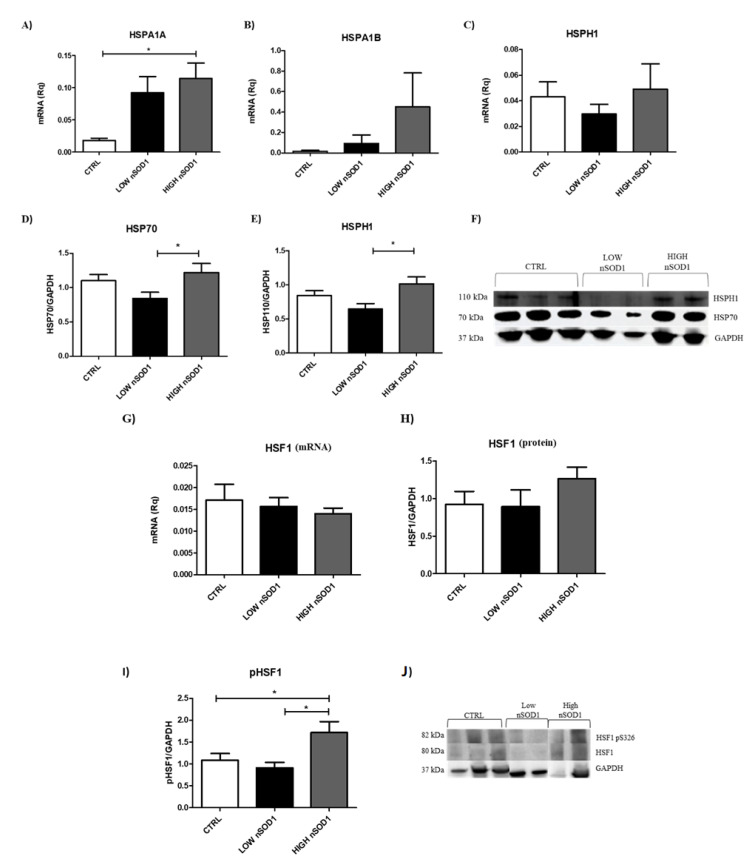
High nSOD1 shows an increase in heat shock proteins. Validation of HSPs. (**A**–**C**) RT-PCR of HSPA1A (CTRL *n* = 8; LOW *n* = 10; HIGH *n* = 7), HSPA1B (CTRL *n* = 7; LOW *n* = 10; HIGH *n* = 7) and HSPH1 (CTRL *n* = 13; LOW *n* = 9; HIGH *n* = 7) in PBMCs of CTRL, low-nSOD1 sALS patients and high-nSOD1 sALS patients. Data were analyzed by ANOVA (number of analyzed groups = 3) followed by Bonferroni post-test. * *p* < 0.05. Levels of HSPA1A and HSPA1B mRNAs are higher in patients with high nSOD1, confirming RNA-seq results, while no significant alterations are observed in HSPH1 mRNA through qPCR. (**D**,**E**) WB analysis for evaluating expression of HSP70s (CTRL *n* = 13; LOW *n* = 10; HIGH *n* = 9) and HSPH1 (CTRL *n* = 12; LOW *n* = 9; HIGH *n* = 11) in sALS PBMCs. Data were analyzed by ANOVA (number of analyzed groups = 3) followed by Bonferroni post-test. * *p* < 0.05. Levels of HSP70 and HSPH1 are higher in patients with high nSOD1 compared to those with low nSOD1. Phosphorylation of HSF1 is increased in high-nSOD1 PBMCs of sALS patients. (**F**) Representative WB membrane for HSP70s and HSHP1. (**G**) RT-PCR of HSF1 mRNA (CTRL *n* = 18; LOW *n* = 10; HIGH *n* = 8) in PBMCs of CTRL, low-nSOD1 sALS patients and high nSOD1 sALS patients. (**H**) WB analysis of HSF1 protein. Their levels do not change. (**I**) WB analysis for the study of HSF1 phosphorylation at serine 326 (CTRL *n* = 13; LOW *n* = 9; HIGH *n* = 10). Levels of phosphorylated HSF1 at S326 are higher in patients with high nSOD1 compared to those with low nSOD1 and to healthy controls. HSF1 Ps326 was normalized to total HSF1. Data were analyzed by ANOVA (number of analyzed groups = 3) followed by Bonferroni post-test. * *p* < 0.05. (**J**) Representative WB membrane for pHSF1 and HSF1.

Since SOD1 has been implicated in the DNA damage response [26], in Supplementary Table S3, we report the DE genes involved in DNA repair processes. KDM4C and TP53TG3D were found to be upregulated in the low-nSOD1 group, and HSPA1A, HSPA1B, FILIP1L and PTGFRN were found to be upregulated in the high-nSOD1 group. We found heat shock proteins (HSPs) to be upregulated in RNA-seq in high-nSOD1 patients (Figure 5; Supplementary Table S2). Of note, HSPs play an important role in the maintenance of both protein homeostasis and DNA integrity [27]. Thus, we evaluated HSPA1A, HSPA1B (both transcribed by HSP70 genes) and HSPH1 mRNA expression (Figure 6A–C) and protein levels in PBMCs (Figure 6D–F). The RNA levels of HSPA1A and HSPA1B mRNAs were significantly increased in high-nSOD1 patients compared to those in the low-nSOD1 group, while no significant alterations in the HSPH1 gene were observed through RT-PCR. In the Western blot analysis, the levels of HSP70 and HSPH1 were higher in patients with high nSOD1 compared to those with low nSOD1. We also measured the levels of the transcription factor HSF1, which regulates the expression of heat shock genes [28]. When triggered, HSF1 becomes trimerized and phosphorylated, and then it translocates into the nucleus, where it binds to conserved heat shock-responsive DNA elements (HSEs) to upregulate genes coding for HSPs [28]. The levels of both HSF1 transcript and protein were unaltered (Figure 6G,H), while the phosphorylation of HSF1 protein at S326 was higher in patients with high nSOD1 compared to both low-nSOD1 patients and healthy controls (Figure 6I,J). These results show the greater activation of HSP70 and HSPH1 in PBMCs of patients with high nSOD1 distribution. 

**Figure 7:**
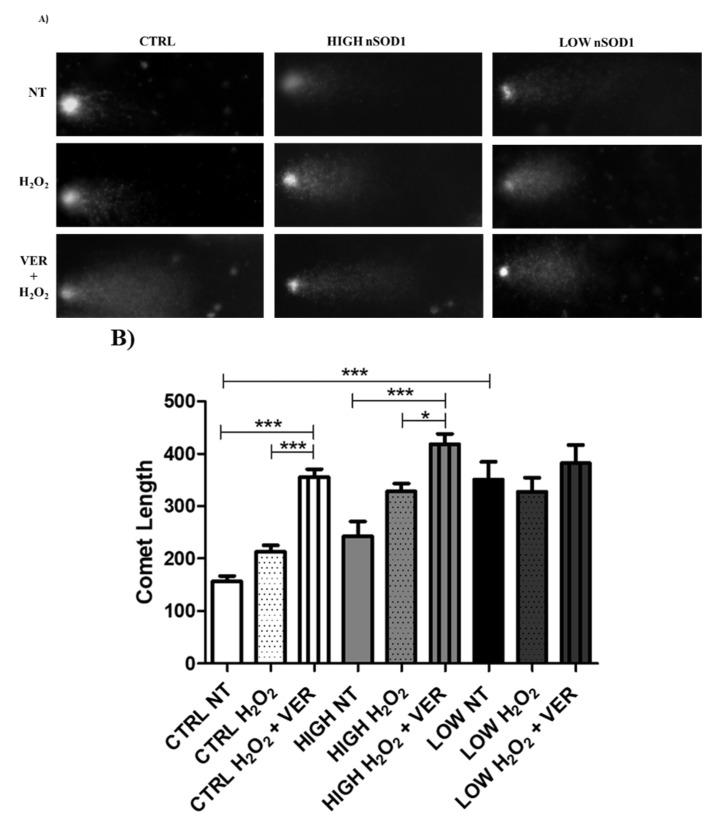
HSP70 is involved in DNA damage protection in PBMCs of sALS patients. (**A**) Protective role of nuclear SOD1 against DNA damage in PBMCs. Controls (*n* = 2) and high- (*n* = 2) and low- nSOD1 (*n* = 2) PBMCs underwent basal evaluation (NT: not treated) or H_2_O_2_ (5 min; 500 μM) treatment followed by recovery or H_2_O_2_ (5 min; 500 μM) + VER (1 h; 50 μM) treatment followed by recovery. (**B**) Comet assay quantification by comet length. Data were analyzed by ANOVA (number of analyzed groups = 3) followed by Bonferroni post-test. * *p* < 0.05 and *** *p* < 0.001. Mean of analyzed cells for each sample *n* = 15.22. An increased comet length, indicating DNA damage, was observed in basal conditions in patients with low nSOD1 compared to controls. Treatment with H_2_O_2_ (5 min; 500 μM) + VER (1 h; 50 μM) visibly increased DNA damage compared to both cells in basal conditions and cells treated with H_2_O_2_ (5 min; 500 μM) only in healthy controls. In high-nSOD1 patients, no significant variation was detected when comparing basal cells and cells treated with H_2_O_2_ (5 min; 500 μM), meaning that upregulation of HSP70 restored normal DNA repair during recovery phase. Inhibition of HSP70 with VER (1 h; 50 μM) prevented the re-establishment of this mechanism. In the end, in low-nSOD1 PBMCs, no significant variation in terms of comet length was observed.

To demonstrate the protective role of SOD1 and its relationship with HSP70 in the nucleus, we performed a comet assay in PBMCs of controls and high- and low-nSOD1 sALS patients (Figure 7A,B). Comet length was measured for cells maintained in basal conditions or treated with H_2_O_2_ (5 min of 500 μM H_2_O_2_) followed by 30′ of stress recovery or, alternatively, subjected to 1 h of 50 μM VER (HSP70 inhibitor) treatment plus 5′ of 500 μM H_2_O_2_ treatment followed by 30 min of stress recovery. An increased comet length, indicating DNA damage, was observed in basal conditions in patients with low nSOD1 compared to controls (Figure 7A,B (*** *p* < 0.001)), while this was not evident in PBMCs of patients with high nSOD1 (Figure 7A,B). In healthy subjects, treatment with H_2_O_2_ (5′; 500 μM) + VER (1 h; 50 μM) visibly increased DNA damage compared to both cells in basal conditions and control cells treated with H_2_O_2_ alone. This indicates that HSP70 is implicated in the recovery phase, and its inhibition strongly affects dsDNA repair. In high-nSOD1 patients, as in the healthy controls, no significant variation was detected when comparing basal cells and cells treated with H_2_O_2_ (5 min; 500 μM), meaning that the upregulation of HSP70 restored normal DNA repair during the recovery phase. On the contrary, the inhibition of HSP70 with VER (1 h; 50 μM) prevented recovery from damage, as expected. Lastly, in low-nSOD1 PBMCs, no significant variation in terms of the comet length was observed in any of the treatments (Figure 7A,B). This suggests that HSP70 is not sufficiently expressed, and its inhibition does not contribute to DNA damage increase, which was present regardless of the treatment. 

## 4. Discussion

Based on our previous reports [3,12], sALS patients can be classified into two groups based on the expression levels of nuclear SOD1: high nSOD1 and low nSOD1. To explore the molecular alterations that distinguish sALS patients classified as high and low nSOD1, we performed transcriptome profiling with differential gene expression analysis in PBMCs. 

Our results clearly indicate that the RNA expression profile of high-nSOD1 patients is more similar to that of healthy controls compared to the low-nSOD1 subgroup. 

These data are consistent with our previous studies demonstrating molecular differences between the two patient subgroups [3,12]. This is also supported by the greater number of dysregulated coding and noncoding transcripts that were found in low-nSOD1 cells, indicating more dysregulated cellular processes in these patients.

For a better understanding of the transcriptomic–phenotypic relation that may exist in ALS, we evaluated the concentration of nSOD1 in patients according to their age and demonstrated that nSOD1 decreases with aging.

Since SOD1 may act as a protective protein in the nucleus by preventing DNA damage [2], we investigated the profiles of genes involved in DNA repair processes in the two subgroups of patients. Gene database/literature research was performed, and we identified six genes implicated in genomic stability maintenance (Supplementary Table S3). KDM4C and TP53TG3D were found to be upregulated in the low-nSOD1 group. The former is not directly involved in DNA integrity maintenance, but its expression is induced by p53, a central player in cellular DNA damage responses [29]. KDM4C has been found to be associated with chromatin during mitosis. This association is accompanied by a decrease in the mitotic levels of H3K9me3. Moreover, research has shown its implication in cell senescence [30,31]. In the high-nSOD1 group, there were four upregulated genes related to DNA damage and repair mechanisms. The most upregulated genes were HSPA1A and HSPA1B, both belonging to the HSP70 family. These HSPs, known for their role in the maintenance of protein homeostasis [32], are also induced in response to DNA-damaging agents, facilitating DNA repair [33,34]. In addition, in the high-nSOD1 group, FILIP1L was upregulated. This is a gene that shares similarities to the bacterial SbcC ATPase DNA repair protein [35]. The expression of FILIP1L is dependent on ATM/ATR [36], which was found to be activated in SH-SY5Y exposed to oxidative stress [3]. The fourth upregulated gene in this group was PTGFRN. It has been demonstrated that the downregulation of this gene leads to reduced DNA damage sensing [37]. These results suggest that patients with a higher concentration of nSOD1 have enhanced activation of genes involved in DNA integrity maintenance, corroborating our previous study demonstrating more damaged DNA in low-nSOD1 patients. 

Interestingly, HSPA1A is overexpressed in high-nSOD1 patients. HSPA1A folding activity can be regulated by other chaperones that also act as nucleotide exchange factors, such as HSPH1, HSPH2 and HSPH3 [38]. Notably, in high-nSOD1 samples, we further detected the upregulation of HSPH1 mRNA, and moreover, we also confirmed HSP70 and HSPH1 protein upregulation. In the low-nSOD1 group, we did not detect any alterations in the expression of HSPA1A, HSPH1 or any other heat shock protein transcript levels. Several studies have additionally addressed the direct involvement of HSPs in the DNA damage response [33,39,40]. They can regulate DNA repair signaling pathways and are required to stabilize core components of DNA repair mechanisms. Altered expression levels of HSPs could lead to impaired DNA damage detection as well as delayed repair [41]. HSP70s, which include HSPA1A and HSPA1B, strongly accumulate in the nucleus upon formation of DNA single-strand breaks caused by metabolic reactive oxygen species production. As a whole, these observations strongly support our hypothesis that high levels of nSOD1 can modulate the activity of factors involved in DNA damage protection. In fact, we observed signs that upon oxidative stress, only healthy controls and high-nSOD1 cells were able to restore DNA integrity. However, when we inhibited the activity of HSP70s, DNA damage was increased in controls and in high-nSOD1 PBMCs. 

The detection of altered S100B transcript levels in PBMCs of low-nSOD1 patients might explain their worse prognosis. In fact, S100B is a Ca^2+^ binding protein involved in a vast number of intracellular and extracellular effects in the brain [42]. It is passively released from damaged and/or necrotic cells, and it increases oxidative stress by binding to RAGE (receptor for advanced glycation end products) [42]. It has already been demonstrated that both human and mouse ALS spinal cord tissues display increased transcript and protein levels of both RAGE and S100B [43]. Our results indicate that S100B transcript upregulation is solely present in low-nSOD1 PBMCs, and thus, it might exert its toxic activity on cell survival and aggravate the ALS pathology exclusively in this subgroup. 

Epigenetic modifications and the resulting effects on gene expression regulation factors are crucial for the integrity of the neuronal population, as demonstrated for repressor element 1-silencing transcription factor (REST) in aging [44]. We observed a strong difference in gene expression in the two sALS subgroups. Thus, we also evaluated the amount of H3K27me3, an important marker of gene expression repression [25]. In fact, in high-nSOD1 PBMCs, trimethylation is greater than in the low-nSOD1 group, in accordance with the lower number of DE genes found in these patients. In the low-nSOD1 group, trimethylation is much lower, partially explaining the higher number of DE genes that emerged from our analysis. However, H3K27me3 is not a specific substrate of KDM4C [45], which was upregulated in low-nSOD1 patients. The data related to histone methylation need to be further evaluated to highlight the effect of SOD1 subcellular localization on epigenetic modifications. In fact, these modifications, responsible for maintaining the intact state of chromatin and its epigenetic features, could be affected by nSOD1. When nSOD1 is low, the balance is lost, and strong deregulation is observed. 

## 5. Conclusions

In conclusion, this work highlights that high nSOD1 levels activate HSP70 family genes in PBMCs, positively affecting the survival of sALS patients. This could possibly explain the differences in disease severity and duration observed in the two patient subgroups [3,12]. Further investigations are needed to better corroborate the proposed mechanism, its cellular implications and its regulation. 

## Figures and Tables

**Table 1:** Baseline characteristics of subjects recruited for this study.

**Patient**	**Age**	**Sex**	**Onset**	**SOD1/PCNA**
M53	53	M	Spinal	1.935
F69	69	F	Spinal	1.673
M69	69	M	Spinal	2.021
F69	69	F	Spinal	1.807
F64	64	F	Spinal	2.903
M58	58	M	Spinal	4.456
M44	44	M	Spinal	3.395
F58	58	F	Bulbar	1.566
F66	66	F	Spinal	1.195
M65	65	M	Bulbar	1.319
M68	68	M	Spinal	1.275
F71	71	F	Spinal	0.718
F74	74	F	Bulbar	0.319
F70	70	F	Spinal	0.539
M66	66	M	Spinal	1.149
M72	72	M	Spinal	0.624
F89	89	F	Bulbar	0.492
F76	76	F	Bulbar	0.222
**Control**	**Age**	**Sex**	**Onset**	**SOD1/PCNA**
M68	68	M	/	2.334
M64	64	M	/	1.300
M63	63	M	/	0.909
M61	61	M	/	4.203
M60	60	M	/	2.088
F58	58	F	/	1.031
M57	57	M	/	0.404
M56	56	M	/	1.692
F51	51	F	/	0.151
M51	51	M	/	0.635
M46	46	M	/	3.075
M45	45	M	/	2.955

Baseline characteristics of subjects recruited for this study. sALS: male = 44.4%; female = 55.6%; age (M ± SD) 66.72 ± 9.63. CTRL: male = 83.3%; female = 16.7%; age (MC ± SD) 56.67 ± 7.19. SOD1 and PCNA1 (reference nuclear protein) ratio was calculated to separate high- and low-nSOD patients. Threshold was set at 1.4.

**Table 2:** Differentially expressed genes reported according to deregulation and gene biotype.

	Low nSOD1			High nSOD1		
	mRNA	lncRNA	Other	mRNA	lncRNA	Other
**UP**	20	9	11	12	2	3
**DOWN**	15	5	2	3	2	3
**Subtotal**	35	14	13	15	4	6
**Total**	62			25		

Differentially expressed genes in each group are classified in relation to regulation (up or down). In the “lncRNA” column, only antisense RNA and long intergenic noncoding RNA (lincRNA) are considered due to their roles in gene expression modulation. In the “Other” column, different biotypes of noncoding RNAs are reported (processed pseudogenes, processed transcripts), but they are not the focus of this work.

## Data Availability

The RNA-sequencing datasets for this manuscript are publicly available, as they are linked to the GEO repository (GSE183204) and at 10.5281/zenodo.5361997.

## Supplementary Materials

The following are available online at https://www.mdpi.com/article/10.3390/cells11020293/s1, Figure S1: SOD1 expression in nucleus; Figure S2: Uniquely mapped read count per each sample; Figure S3: Transcript biotypes; Figure S4: Dot plot of KEGG pathway analysis; Table S1: Differentially expressed transcripts in low-nSOD1 group; Table S2: Differentially expressed transcripts in high-nSOD1 group; Table S3: Genes involved in DNA damage; Table S4: Oligonucleotide sequences.
