# Supplementary material for: RNA Molecular Signature Profiling in PBMCs of Sporadic ALS Patients: HSP70 Overexpression Is Associated with Nuclear SOD1
Source: Cells. 2022 Jan 15;11(2):293. doi: 10.3390/cells11020293 (PMC8774074; doi:10.3390/cells11020293)
Supplement: Supplementary file 1 [file cells-11-00293-s001.zip › cells-1504722-supplementary_.pdf]

Supplementary data

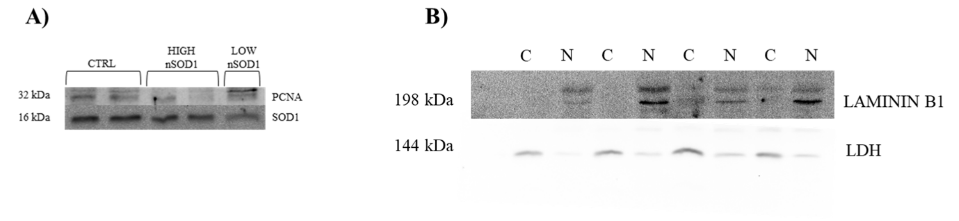

Supplementary Figure S1. A) Representative WB membrane of SOD1 expression in nucleus. WB quantification of nSOD1 distribution in nuclei of PBMCs isolated from healthy controls (CTRL) and sALS patients normalized to PCNA. B) Representative WB membrane showing the absence of cross-contamination between the two subcellular fractions. Nuclear fractions were tested for the presence of the cytoplasmic protein LDH (144 kDa) while the presence of cytoplasmic contamination in nuclear fractions was tested using the nuclear protein LAMININ B1 (144 kDa). The level of cross-contamination between the two subcellular fractions is very low; hence, no significant cross-contamination occurs between the two extracts. C= cytoplasmic fraction; N= nuclear fraction.

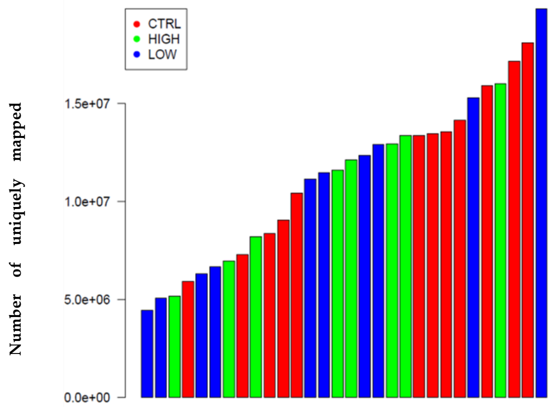

Supplementary Figure S2. Uniquely mapped reads count per each sample. Red bars represent CTRL, green bars represent high nuclear SOD1 samples, and blue bars represent low nuclear SOD1 samples.

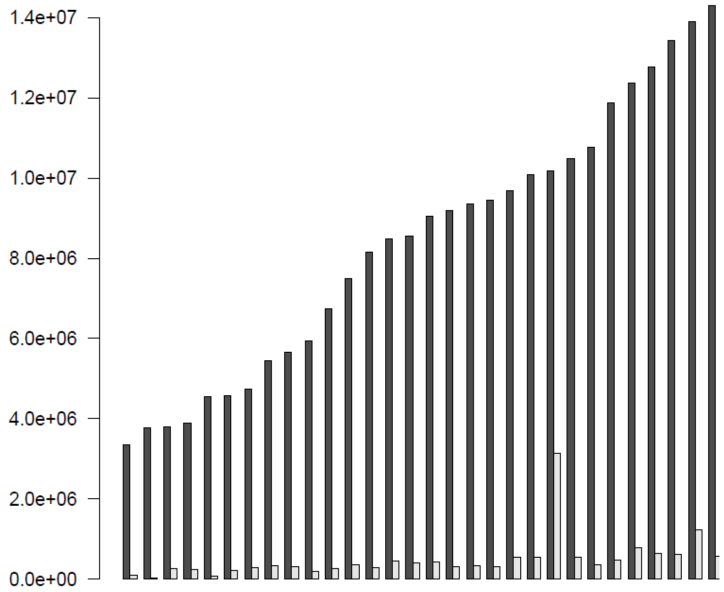

Supplementary Figure S3. Transcripts biotype. Number of detected transcripts per sample for coding (dark grey bars) and non-coding RNAs (light grey bars).

Supplementary Table S3. Genes involved in DNA damage process in low nSOD1 and in high nSOD1 are reported together with their Fold Change.

Low nSOD1		High nSOD1	
**Gene**	**Fold Change**	**Gene**	**Fold Change**
KDMC4	7.52	HSPA1B	3.99
TP53TG3D	2.53	HSPA1A	2.00
		FILIPL1	1.79
		PTGFRN	1.41

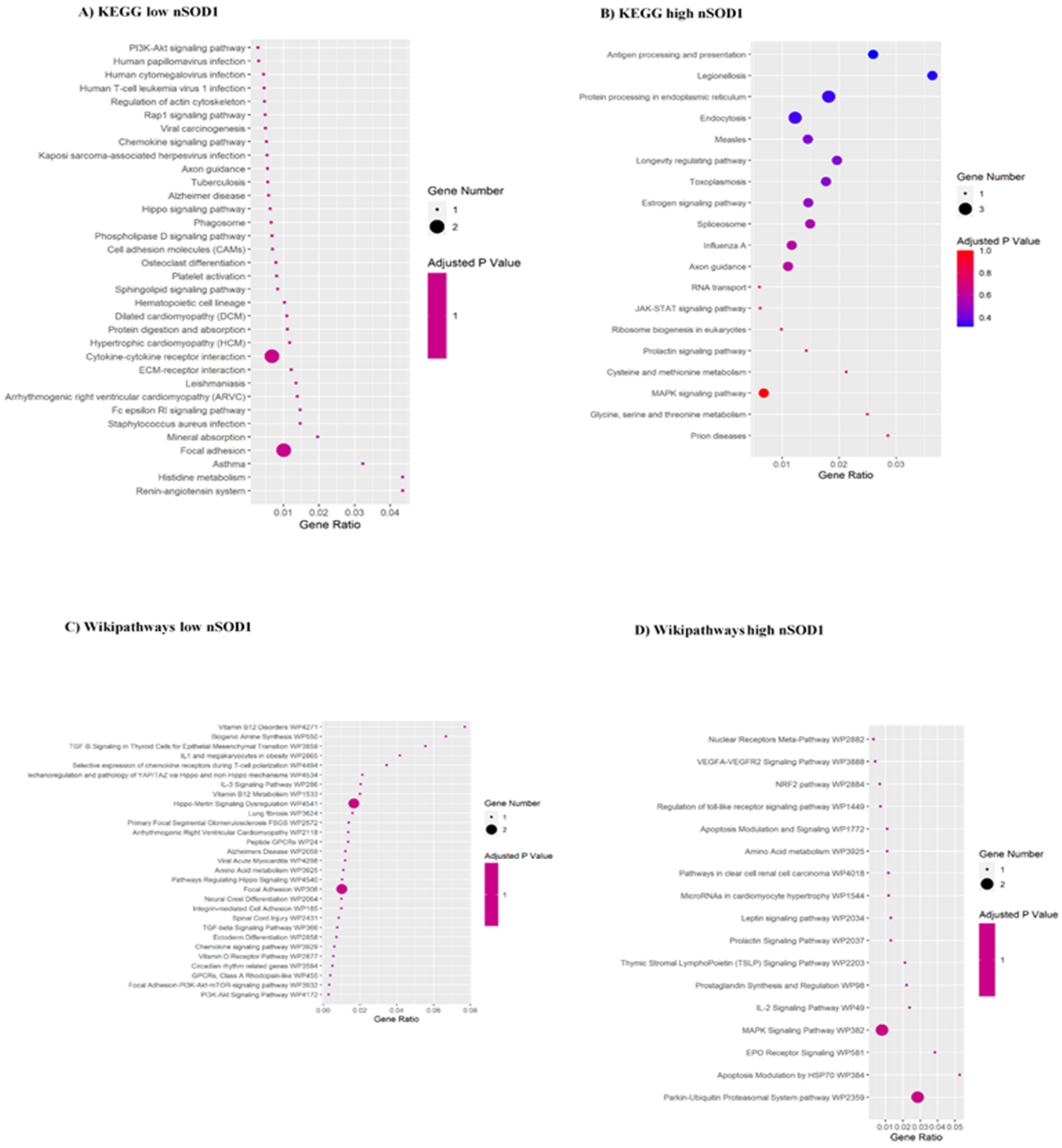

Supplementary Figure S4. Dot Plot of KEGG pathway analysis. Dot plot shows the dysregulated KEGG and Wikipathways pathways (FDR <0.1) enriched for low (A, C) and high (B, D) nSOD1 patients. The size of the dot is based on gene count enriched in the pathway, and the colour of the dot shows the pathway enrichment significance. Gene ratio: the ratio of resulting DE genes correlated to a KEGG pathway to the number of annotated genes per KEGG pathway in the database. The p-value is computed from the Fisher exact test which is a proportion test that assumes a binomial distribution and independence for probability of any gene belonging to any set.

Supplementary Table S4. Oligonucleotides used for RT-PCR validation of RNA-seq DE genes.

Transcript	Sequence
GAPDH Forward	5'-ATGGAAATCCCATCACCATCTT-3'
GAPDH Reverse	5'-CGCCCCACTTGATTTTGG-3'
HSPA1A Forward	5'-CGACCTGAACAAGAGCATCA-3'
HSPA1A Reverse	5'-AAGATCTGCGTCTGCTTGGT-3'
HSPA1B Forward	5'-CCGAGAAGGACGAGTTTGAG-3'
HSPA1B Reverse	5'-GCAGCAAAGTCCTTGAGTCC-3'
HSPH1 Forward	5'-CTCCCAAAGTGCTGGGATTA-3'
HSPH1 Reverse	5'-CATCTTCACCCAGGAAGCAT-3'
HSF1 Forward	5'-GACATAAAGATCCGCCAGGA-3'
HSF1 Reverse	5'-CTGCACCAGTGAGATCAGGA-3'
